# 3D-FISH analysis reveals chromatid cohesion defect during interphase in Roberts syndrome

**DOI:** 10.1186/s13039-014-0059-6

**Published:** 2014-09-30

**Authors:** Celine Dupont, Martine Bucourt, Fabien Guimiot, Lilia Kraoua, Daniel Smiljkovski, Dominique Le Tessier, Camille Lebugle, Benedicte Gerard, Emmanuel Spaggiari, Pierre Bourdoncle, Anne-Claude Tabet, Brigitte Benzacken, Jean-Michel Dupont

**Affiliations:** Unité fonctionnelle de Cytogénétique-Département de Génétique- APHP, Hôpital Robert Debré, 48 Bd Sérurier, 75935 Paris, France; Laboratoire de Fœtopathologie- APHP, Hôpital Jean Verdier, Bondy, France; Service de Biologie du Développement- APHP, Hôpital Robert Debré, Paris, France; Université Paris Diderot Sorbonne Paris Cité, UMR 1141, F-75019 Paris, France; Unité fonctionnelle de Génétique moléculaire - Département de Génétique- APHP, Hôpital Robert Debré, Paris, France; Génomique, Epigénétique et Physiopathologie de la Reproduction, U1016 INSERM-UMR 8104 CNRS (Institut Cochin), Université Paris Descartes, Faculté de Médecine, Paris, France; Laboratoire de Cytogénétique- APHP, Hôpitaux Universitaires Paris Centre, Paris, France; Institut Cochin, Plateforme d’imagerie cellulaire, Paris, France; Service d’Histologie, Embryologie et Cytogénétique, Biologie de la Reproduction- APHP, Hôpital Jean Verdier, Bondy, France; UFR-SMBH, Paris, XIII France

**Keywords:** Cohesinopathy, *ESCO2*, Heterochromatin, Limb development

## Abstract

**Background:**

Roberts syndrome (RBS) is a rare autosomal recessive disorder mainly characterized by growth retardation, limb defects and craniofacial anomalies. Characteristic cytogenetic findings are “railroad track” appearance of chromatids and premature centromere separation in metaphase spreads. Mutations in the *ESCO2 (establishment of cohesion 1 homolog 2)* gene located in 8p21.1 have been found in several families. ESCO2, a member of the cohesion establishing complex, has a role in the effective cohesion between sister chromatids. In order to analyze sister chromatids topography during interphase, we performed 3D-FISH using pericentromeric heterochromatin probes of chromosomes 1, 4, 9 and 16, on preserved nuclei from a fetus with RBS carrying compound heterozygous null mutations in the *ESCO2* gene.

**Results:**

Along with the first observation of an abnormal separation between sister chromatids in heterochromatic regions, we observed a statistically significant change in the intranuclear localization of pericentromeric heterochromatin of chromosome 1 in cells of the fetus compared to normal cells, demonstrating for the first time a modification in the spatial arrangement of chromosome domains during interphase.

**Conclusion:**

We hypothesize that the disorganization of nuclear architecture may result in multiple gene deregulations, either through disruption of DNA *cis* interaction –such as modification of chromatin loop formation and gene insulation - mediated by cohesin complex, or by relocation of chromosome territories. These changes may modify interactions between the chromatin and the proteins associated with the inner nuclear membrane or the pore complexes. This model offers a link between the molecular defect in cohesion and the complex phenotypic anomalies observed in RBS.

**Electronic supplementary material:**

The online version of this article (doi:10.1186/s13039-014-0059-6) contains supplementary material, which is available to authorized users.

## Background

Roberts Syndrome (RBS, OMIM #268300) is a rare autosomal recessive disorder first described by John Roberts in 1919. This multiple congenital anomaly syndrome is characterized by cleft lip and palate, nose and ears anomalies, facial hemangioma, hypertelorism, microcephaly, curly silvery blond hair, reductional limb defects leading to oligodactyly or tetraphocomelia, polycystic or dysplastic kidneys, congenital heart defects, enlarged male genitalia, severe growth retardation and intellectual deficiency [1]. Vega *et al*. identified homozygous or compound heterozygous *ESCO2 (establishment of cohesion 1 homolog 2)* mutations in 15 families with RBS [2]. *ESCO2* is one of the two human orthologs of a yeast gene Eco1/Ctf7 involved in sister chromatid cohesion. The C-terminal domain of the protein is evolutionarily conserved and harbors an acetyltransferase (AT) activity [2]. To date, more than 30 different mutations have been described [2–8], most of which result in protein truncation with an absence of evidence for genotype-phenotype correlation [7]. RBS is characterized by a “railroad-track” appearance of metaphase chromosomes, due to premature centromere separation (PCS), a phenomenon also described as heterochromatin repulsion [1]. PCS is more prominent in the pericentromeric, heterochromatic regions of chromosomes 1, 9 and 16, in the p arm of acrocentric chromosomes, and in Yq heterochromatin.

During interphase, chromosomes are organized in the nucleus into individual “chromosome territories” (CTs) that are non-randomly scattered [9]. The position of CT depends on their volume [10] and on gene density [11]. Nuclear architecture and chromatin organization during interphase probably contributes to gene expression [9]. Abnormal spatial organization of the CTs in interphase was reported in tumor cells carrying chromosome translocations [12], in epilepsy [13], in laminopathies [14], and in ICF syndrome [15,16].

To get new insights into the relationship between *ESCO2* mutations, cytogenetic anomalies and clinical features in Roberts syndrome, we performed 3D-FISH using pericentromeric heterochromatin (PH) probes of chromosome 1, 4, 9 and 16 on fibroblasts and cytotrophoblasts nuclei of a patient with molecularly defined RBS.

## Results

### Patient data

A 27-year-old mother was referred for cytogenetic prenatal diagnosis after abnormal ultrasound findings (tetraphocomelia, hygroma and facial dysmorphism) at eleven weeks of gestation. Ultrasound scan at 16 weeks confirmed symmetrical limb defects with absent forearms, presence of only one bone in each leg and echogenic bowels. Death occurred in utero at 18 weeks. Parents were healthy, unrelated, from African origin.

**Figure 1:**
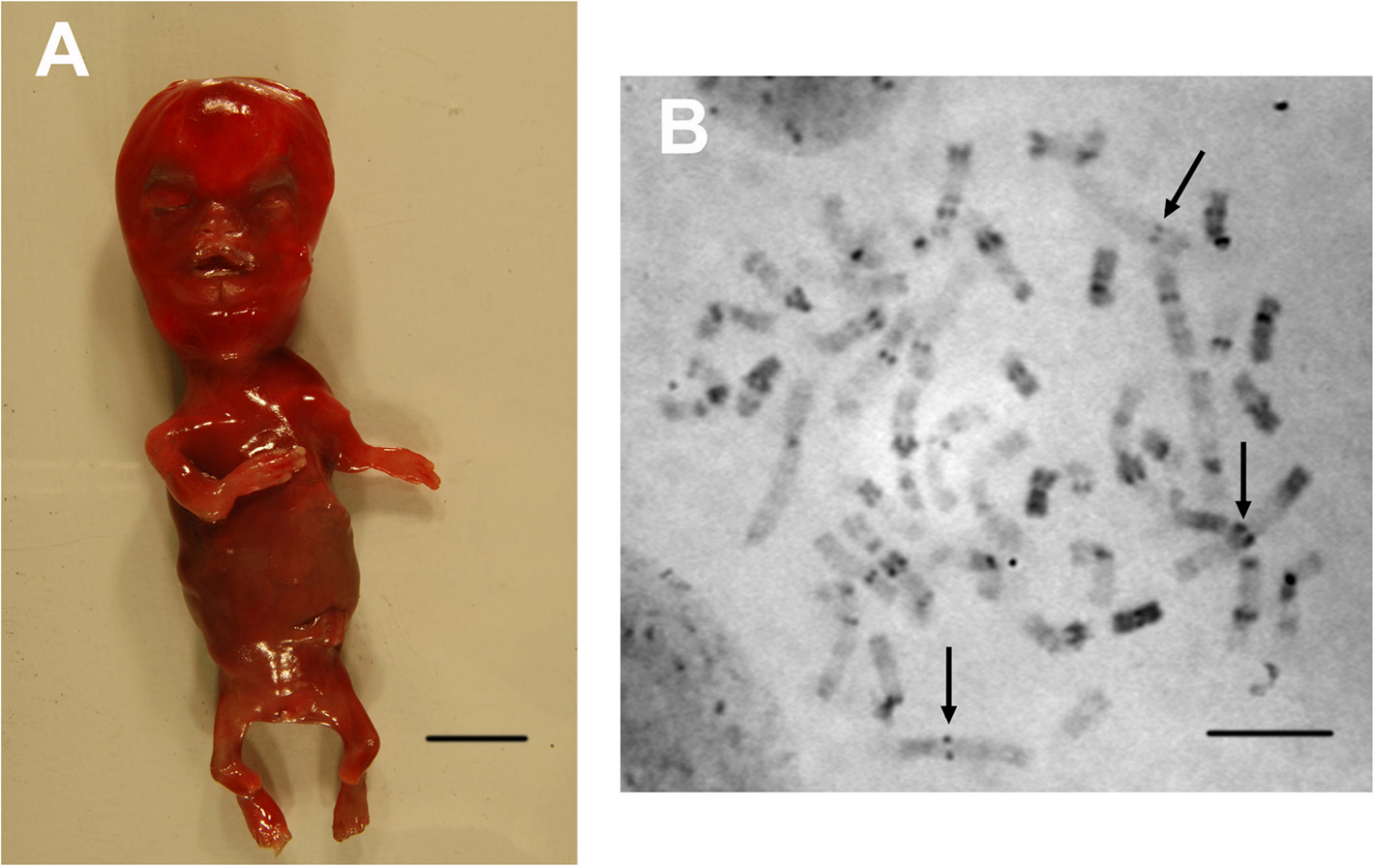
**Clinical and cytogenetical description of the fetus. (A)**: Fetus of 18 weeks gestation with multiple congenital anomalies: hypertelorism, micrognathia, tetraphocomelia and oligodactyly. Scale bar = 2 cm **(B)**: C-banded metaphase chromosomes from the affected fetus showing the pathognomonic cytogenetic anomaly in Roberts Syndrome: chromosomes with premature centromere separation: PCS (black arrows) and heterochromatin puffing. Scale bar = 5 μm.

The female fetus weighted 70 gr (<<5^th^ centile), and has a crown-rump length of 11.5 cm (<<5^th^ centile). He had tetraphocomelia (Figure 1A). X ray survey showed bilateral absence of radii, ulnae, and fibulae, oligodactyly (4 fingers in each hand) and hypoplasia of the fifth toes. He had median frontal bone defect, small, flat nose with hypoplastic nasal bone, hypertelorism, exophthalmia, short philtrum, adhesion of upper lip to the upper gum, and low-set ears. There were no visceral malformations. Histological examination was not contributive. R- and G-banded karyotype from a chorionic villus sample was 46,XX. In skin fibroblasts, C-banded metaphases showed premature centromere separation and puffing of the heterochromatin (Figure 1B).

### Mutational analysis of ESCO2

Analysis of ESCO2 gene in our patient revealed the presence of two compound heterozygous mutations: one mutation is a substitution of an Adenine for a Guanine base (c.1131 + 1G > A) and had been previously described [3]; the second mutation is a 4-bp deletion overlapping the end of exon 4 and the beginning of intron 4 (c.954_955 + 2delAAGT) (Additional file 1: Figure S1). The first mutation (c.1131 + 1G > A) is a splice-site mutation in intron 6 resulting in a skipping of exon 6 (r.1014_1131del118) and causing a premature termination of translation (p.R338fs*17) with no evidence of a normal transcript [3]. The second mutation (c.954_955 + 2delAAGT) has never been described before; it interrupts the splice donor site of intron 4 resulting probably in skipping of exon 4, causing also a premature termination of translation of the ESCO2 protein (p.ASP288Phefs*22). Both mutations have to be considered as null alleles, with no functional ESCO2 protein. Unfortunately, the parents were not available for molecular study of ESCO2.

### 3D FISH: Behavior of pericentromeric heterochromatin of chromosome 1 in interphase RBS cells

**Figure 2:**
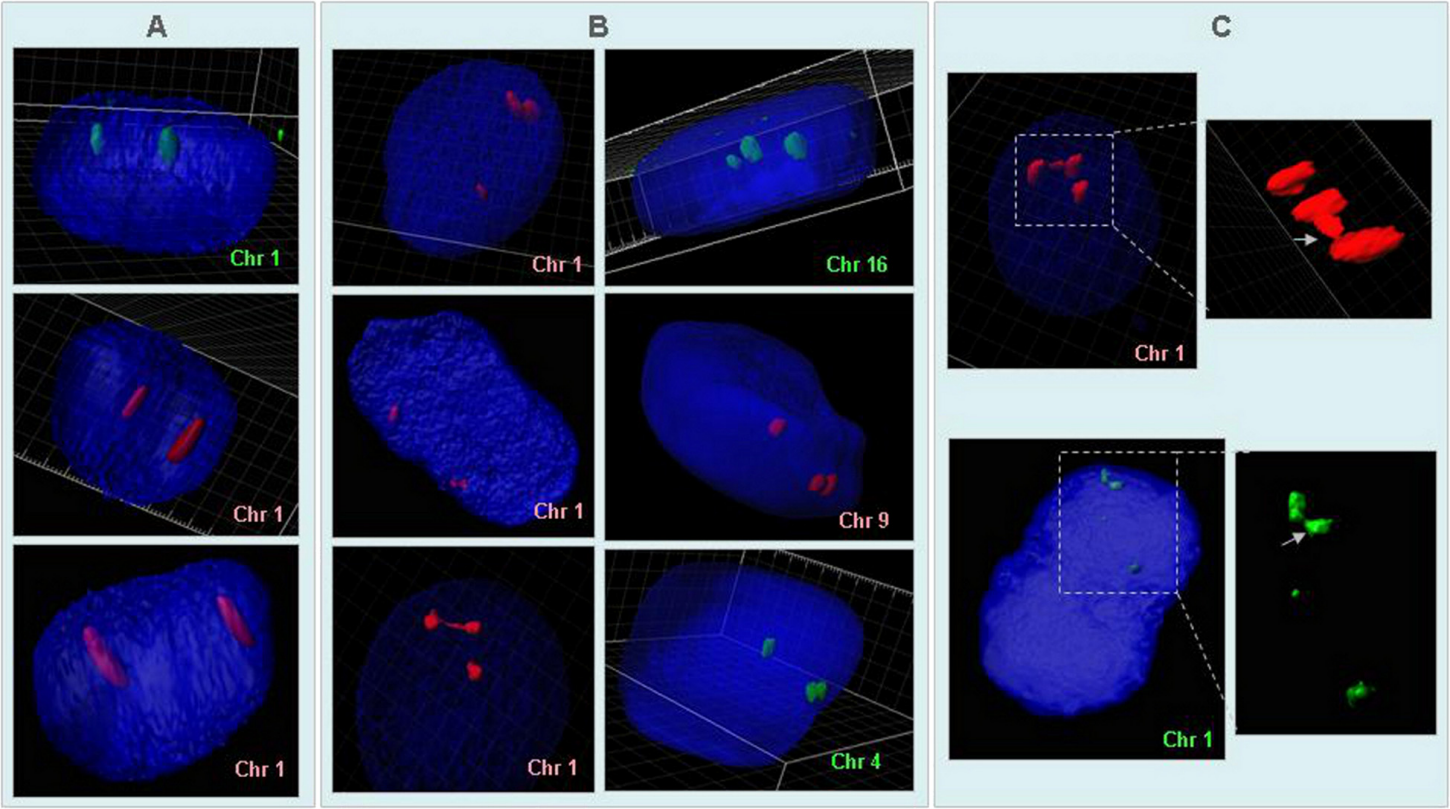
**3D FISH of Roberts sub-chromosomal domain territories.** Views of Imaris® reconstructions of trophoblasts and fibroblasts nuclei after three-dimensional FISH with probes for PH1 (Chr1), centromeric régions of chromosome 4 (Chr4), 9 (Chr9) and 16 (Chr16). Cells were counterstained with DAPI (blue). **(A)** Control cells hybridized with PH1 probe (trophoblasts: red probe and a fibroblast: green probe). **(B)** RBS cells with split signal of one or two territories (chr 1, chr 4, chr 9 and chr 16). **(C)** Focus on split spots of PH1 showing the bridge between the two signals (arrows).

Using 3D FISH we showed that approximately 90% of RBS fibroblasts (7/8) and trophoblasts (23/26) nuclei had a split configuration of one or two PH1 signals (Figure 2A). Pericentromeric heterochromatin of chromosomes 4, 9 and 16 was also split during interphase (Figure 2B). This was not observed in control cells.

In some of the most widely split PH1 sub-territories, a fluorescent signal bridging both chromatids was observed (Figure 2C) that could correspond to intertwined sister DNA molecules.

### 3D FISH: Relocation of PH of chromosome 1 within RBS cells

**Figure 3:**
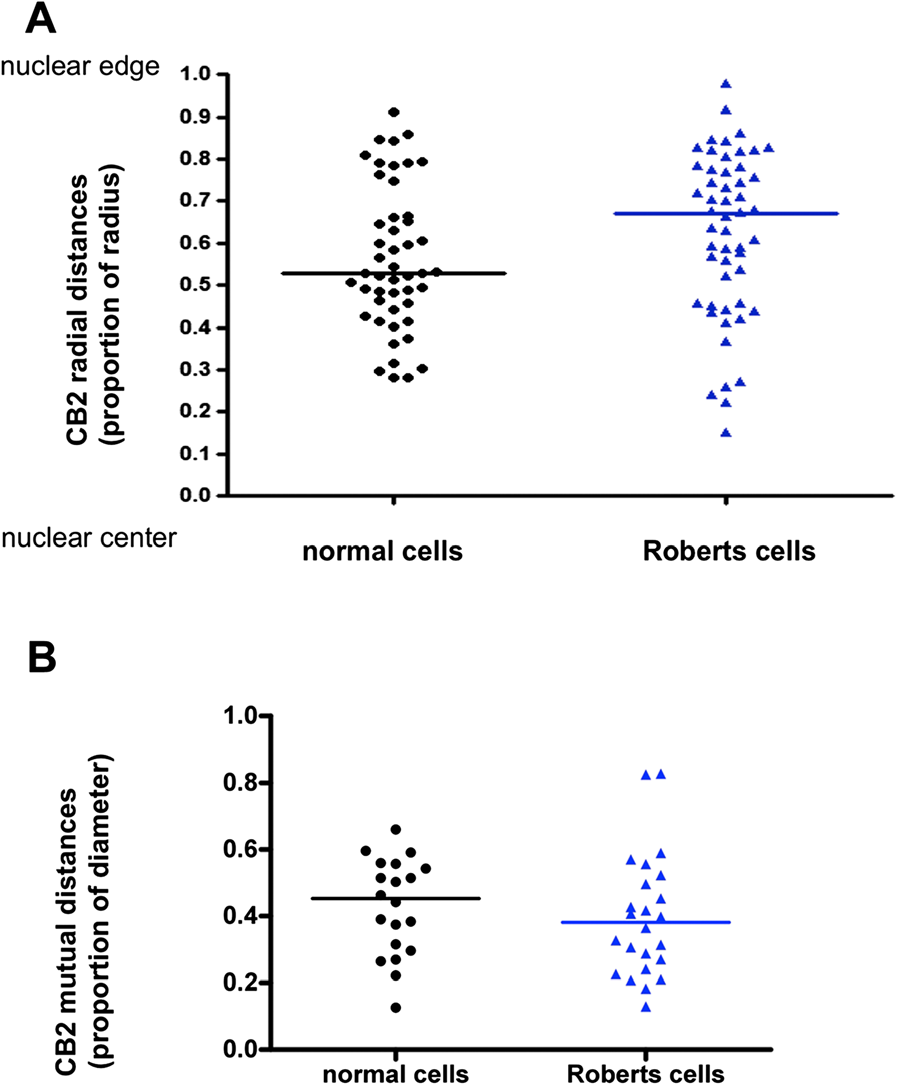
**Statistical representation of CB2 radial and mutual positions. (A)**: Distribution of each PH1 (=CB2 probe) radial position of normal cells (black rings) and Roberts cells (blue triangles). The median was calculated both for normal cells (M^r^n, black line) and Roberts cells (M^r^R, blue line). Radial distances are expressed as a proportion of the radius. M^r^n = 0.5285 and M^r^R = 0.6700: Wilcoxon test was significant (alpha = 0.05) with a p value of 0.0018 showing a significant relocation of PH1 territory towards the edge of the nucleus in Roberts cells. **(B)**: Distribution of CB2 mutual position of normal and Roberts cells. The median was calculated both for normal cells (M^m^n,black line) and Roberts cells (M^m^R, blue line). Mutual distances are expressed as a proportion of the diameter. M^m^n = 0.4527 and M^m^R = 0.3813: Wilcoxon test was significant (alpha = 0.05) with a p value of 0.0829 showing a significant rapprochement of homologous PH1 territories in Roberts cells.

Statistical investigations were performed on PH of chromosome 1 with CB2 probe. Difference in the radial position of PH1 was observed between normal and RBS cytotrophoblasts. Twenty-six RBS nuclei (52 PH1 territories) and 24 control nuclei (48 PH1 territories) were analyzed from cytotrophoblast. The distance of PH1 to the center of the nucleus was significantly increased in RBS nuclei (M^r^R = 0.6700) compared to controls (M^r^n = 0.5285) (Figure 3A). This result was interpreted as a localization of PH1 more peripheral in RBS nuclei than in normal cells. There was also a statistically significant difference between normal and RBS cells with respect to mutual PH1 distances (Figure 3B). We observed that in RBS nuclei, both homologous PH1 territories were closer (M^m^R = 0.3813) to each other than in normal cells (M^m^n = 0.4527) (Wilcoxon test). The same analysis was performed on fibroblasts. We were able to analyze 8 RBS nuclei (16 CB2 territories) and 16 control nuclei (31 CB2 territories). No statistically significant difference in the radial position of PH1 was observed between normal and RBS fibroblasts but a general trend towards a more peripheral location of these territories was noticed for RBS fibroblasts (M^r^R = 0.6213 and M^r^n = 0.5562).

## Discussion

Genome architecture and 3D folding of chromatin fibers during interphase has been increasingly associated with human diseases through modulation of gene expression [9]. Mutations of genes responsible for this specific nuclear organization could lead to developmental disorders such as RBS. A characteristic feature of RBS cells is heterochromatin puffing on metaphase spreads [1]. This finding was the first clue towards a cohesin defect in RBS. Puffing may reflect a modification of the conformation of chromatids. We therefore explored the organization of the centromeric heterochromatin during interphase to check whether modified conformation could reflect an intrinsic reorganization of the chromatin. More than 90% of RBS nuclei observed in our study showed PH splitting of chromosomes 1, 4, 9 and 16, which was never observed in control nuclei. PCS, which is also seen in metaphase chromosomes in RBS, has never been reported before in interphase nuclei. These new data imply that the underlying conformational anomaly is not confined to metaphase but exists throughout the whole cell cycle. In RBS, loss of ESCO2 acetyltransferase activity is crucial for proper sister chromatid cohesion [6] and most of the *ESCO2* mutations described (88%) lead to a premature stop codon prior or within the acetyltransferase domain which is most likely the case of both mutations found in the reported RBS patient. This loss of ESCO2 function may lead to weaker cohesion between sisters and to the splitting of PH observed in the nuclei. Moreover it is known that replication of alpha satellite sequences is delayed in RBS cells [17] which could impact chromatin organization, since cohesins are loaded onto DNA during replication. Our 3D-FISH observations favor the handcuff model to explain cohesin-mediated chromosome tethering [18]. In this model, one cohesin ring encircles only one sister chromatid, and both rings are connected by Scc3 and Rad21 subunits to hold sister chromatids together [18]. The cohesion defect could lead to loose juxtaposition of the two chromatids resulting in split FISH signals. Interestingly, many split PH regions show a bridge between the two signals. This mechanical link might be constituted by inter-twinned centromeric DNA, which has been shown to contribute to centromeric cohesion [19]. Moreover, this bridge could be explained by presence of self-interactions in the Scc1/Rad21 subunit [18].

There is evidence suggesting that there is a non-random radial nuclear distribution of CTs and chromosomal sub-regions during interphase. The relative positioning of chromosome domains plays important roles in genome function and gene expression [9]. While the relationship between *ESCO2* mutations and the cellular phenotype is well known, the link between the defect in chromatid cohesion and the clinical symptoms of RBS, remains elusive. Because *ESCO2* mutations are associated with conformational modifications of chromatin, we explored whether these structural changes could be responsible for phenotypic findings through modification of CT arrangement inside the nucleus. From our 3D-FISH experiments, we could demonstrate a significantly more peripheral position of PH1 in RBS cells compared to control cells.

The pattern of embryonic expression of *ESCO2* is consistent with the tissues affected in RBS individuals [7]. A first clue towards the physiopathogenic mechanism of RBS came from the observation of anomalies in cell growth and proliferation. Gordillo and Tomkins [6,20] proposed that defective mitosis and increased cell death might contribute to the reduced growth and developmental phenotype in RBS. Dorsett has suggested that in addition to the proliferation defect, gene expression may be altered in RBS [21]. We hypothesize that abnormal spatial organization of chromosomes domains in interphase nuclei may be the origin, or the consequence of RBS associated chromatin-mediated alterations in gene expression. Previous studies in yeast and *drosophila*, have established that cohesins play a significant role in gene expression by helping in delimiting the boundaries of silenced chromatin domains [22] or by long range activation of homeobox protein genes [23]. Regulation of many genes by cohesin appears to involve the three-dimensional (3D) organization of chromatin [24,25], but the basis of this cohesin function remains poorly understood. Investigations in mammals with chromatin immunoprecipitation (ChIP) experiments have revealed that cohesin binds to the same sites as CCCTC binding factor (CTCF) [26]. CTCF is a zinc finger binding transcription factor known to be an enhancer-blocking transcriptional insulator. Compelling evidence that cohesin is required for the function of CTCF has been obtained from the analysis of various loci [27], such as the cytokine IFN-γ, β-globin, apolipoprotein gene cluster, and IGF2/H19 [28]. The stabilization of chromatin loops formed by CTCF depends on cohesin allowing some *cis* DNA-DNA specific interaction with maternal or paternal alleles [28]. These observations indicate that CTCF and cohesins may control gene expression by mediating local changes in the chromatin conformation, thereby determining which promoters can physically interact with enhancers or other regulatory sequences [28].

For RBS, we suggest that deregulation of cohesin function could promote transcriptional alteration of some developmental genes by the disruption of *cis*-DNA interactions mediated by CTCF-cohesin complex. Mönnich *et al.,* used a zebrafish model of RBS to analyze, by microarray, the expression of genes downstream of *ESCO2* [29]. They showed that *Esco2*-regulated genes were more likely to be involved in cell-cycle or apoptosis [29]. They proved that even modest *Esco2* depletion resulted in strong activation of caspases, in p53/mdm2 upregulation and in massive cell death, in the first day of development. These results confirm the initial hypothesis on the pathophysiology of RBS. To explore how the deficiency in Esco2 affects cohesin’s functions, Whelan *et al.,* generated a mouse harboring a conditional *Esco2* allele. Their experiments showed that Esco2 is a real cell viability factor and explained the cellular RBS phenotype by two possible mechanisms: 1) a reduction of Sororin recruitement caused by reduced Smc3 acetylation in mutants and resulting in inefficient pericentromeric cohesion at S phase- 2) a relocalisation of Shugoshin (Sgo1) to the chromosome arm resulting in a de-protection of centromeric cohesion [30].

The expression of some key genes is probably modified in tissues from RBS patients and could be measured by microarray studies in order to explore how this modification in the spatial arrangement of chromosome affects the transcriptome. We observed a change in spatial conformation of PH1 and there are some genes on chromosome 1, whose deregulation could lead to the RBS phenotype. Indeed, there are genes that play a role in cell growth and division (proliferation) as *CDC73* (1q25), *LEPRE1* (1p34.1), *NRAS* (1p13.2) and *ORC1* (1p32). Some others have an important role in the growth and development of bones: *ALPL* (1p36.12), *COL9A2* (1p33p32), *COL11A1* (1p21), *SKI* (1p36.33) and *TGFB2* (1q41). *KIF1β* (1p36.2) is involved in programmed cell death (apoptosis). Finally *ARID1A* (1p35.3) and LBR (1q42.1) regulate gene activity by chromatin remodeling and *RBM8* (1q21.1) gene is involved in TAR syndrome. All these genes and probably others on different chromosomes could be differentially expressed secondary to the modification in PH1 location or cohesin dysfunction in RBS cells and thus result in abnormal phenotype. Unfortunately, expression analysis by qPCR or expression microarray studies, which requires fresh fetal tissues, could not be performed in this RBS patient.

Because the main silent chromatin domains localize to the nuclear border, the nuclear periphery is generally viewed as an area of “gene silencing”. Silencing is mediated by the tethering of chromatin to the lamina, which provides a distinct regulatory environment through lamina-associated protein and chromatin structure modification [31]. However, the nuclear border is also associated with specific transcriptional activation [32]. Interestingly, the laminar interacting domains are flanked by insulator protein-binding sites like CTCF [33]. We hypothesize that PH1 relocation observed in RBS cells could promote a modification in chromatin-lamina interactions secondary to an altered cohesin function. At this point, we cannot determine whether PH delocalization is a direct consequence of ESCO2 dysfunction, or whether absence of ESCO2, by an unknown mechanism, modifies gene expression, resulting in PH relocalization. PH relocation could be responsible for further alterations in transcriptional activities that finally would explain the RBS phenotype.

## Conclusion

In conclusion, we have shown that *ESCO2* mutations lead to a reorganization of the topology of interphase nucleus, opening new perspectives to enlighten the physiopathology of cohesinopathies.

## Methods

Written informed consent was obtained from the patient for publication of this study and any accompanying images. A copy of the written consent is available for review by the Editor-in-Chief of this journal.

### Molecular analysis of ESCO2

Genomic DNA was isolated from fetal skin sample using the QIAamp DNA Blood Midi kit (QIAGEN GmbH, Hilden, Germany) following the manufacturer’s recommendations. The coding sequences and intron-exon junctions of *ESCO2* (GenBank NM_001017420.2) were directly sequenced. Purified PCR amplification products were sequenced using the BigDye Terminator Cycle Sequencing Kit v.1.1 (Applied Biosystems, Foster City, California, USA) according to the manufacturer’s instructions, and were resolved on an ABI 3130xl automated sequencer (Applied Biosystems). Sequence data were aligned with SeqScape 2.0 software and compared to the published sequences of *ESCO2*.

### Cell culture and preparation

Chorionic villus sampling was performed at 11 WG. Fibroblasts were obtained from Achilles tendon during fetal necropsy. Cytotrophoblasts and fibroblasts cultures were set up following standard protocols. Cells were then washed and stored in PBS. Control cytotrophoblasts and fibroblasts were obtained from two cytogenetically normal fetuses.

### 3D FISH

The protocol was derived from Dupont *et al*. [16]. Air-drying of the preparation was carefully avoided in all experiments to preserve the 3D structure of nuclei. In-house PCR-amplified probe for pericentromeric heterochromatin of chromosome 1 (PH1 = CB2:1q12, satellite II) was labelled with biotin or digoxygenin-16-dUTP by PCR amplification while heterochromatin probes for human chromosome 4 (PH4 = D4Z1, αsatellite), chromosome 9 (PH9 = D9Z3, satellite III) and chromosome 16 (PH16 = D16Z2, αsatellite) were FITC or rhodamin labelled (probes provided by Cytocell®, Cambridge, UK). Hybridization was carried out at 37°C in a humidified chamber for approximately 14 hours. After washing, following standard procedures, digoxigenated and biotinylated probes were detected by anti-digoxigenin-rhodamine or by avidin-FITC conjugate, respectively. All slides were counterstained with 1 μg/ml DAPI.

### Image acquisition and microscopy

3D preparations were examined using a Leica® (Leica microsystem GmbH, Wetzlar, Germany) (TCS SP2 AOBS) confocal microscope equipped with a 63x objective. The camera and the microscope were controlled by the Leica® Confocal Software (LCS). Stacks of optical sections were collected from nuclei showing apparently complete and specific hybridization signals in all channels. Stacks were obtained with an image size of 512×512 pixels. The focus step between sections was 0.5 μm on the average.

For chromosome 1 we analyzed 26 cytotrophoblasts (52 territories) and 8 fibroblasts (16 territories) nuclei from RBS patient. For chromosomes 4, 9 and 16 we only have few nuclei (5 for each probe) to observe and we chose to focus on chromosome 1 to draw statistic data. In parallel, we analyzed 24 cytotrophoblasts (48 territories) and 15 fibroblasts (30 territories) nuclei from a control patient, only for chromosome 1.

Roberts’s cellular phenotype is mainly defined by an abnormal morphology of pericentromeric constitutive heterochromatin, including the large PH1 domain on chromosome 1. This region was selected for further analysis because a specific PCR probe was available in the laboratory [16] and because chromosome 1 was shown to adopt a non ambiguous peripheral position in fibroblast nuclei [10].

### Quantitative evaluation and statistical analysis of data

The 3D reconstructions of PH1-, PH4-, PH9- and PH16-labelled nuclei captured by confocal microscopy were performed using the IMARIS® 7.1 software (Biplane Scientific Software, St. Paul, MN). To calculate distances in an ellipsoid object, the 3D coordinates for each fluorescent object (the nucleus or the PH fluorescent signals) were determined by IMARIS®. The radial position of PH1 ([nuclear center]-to-[PH1] distances) and mutual distances between the two PH1 territories ([PH1]-[PH1] distances) were calculated using a MATLAB® (MathWorks, Natick, MA) script. The measured radial positions of PH1 were normalized to the nuclear radius to allow accurate length comparison. They are presented as a percentage of the nuclear radius [% of R]. In the same way, mutual distances were normalized to the largest nuclear diameter. Statistical analysis of distances was carried out using GraphPad Prism® (GraphPad, La Jolla, CA), using a non-parametric Wilcoxon two-sample rank-sum test comparing median differences in normal and RBS cells. The median was first calculated for normal cells (M^r^n for radial and M^m^n for mutual distances) and then for RBS cells (M^r^R for radial and M^m^R for mutual distances).

## Additional file

Additional file 1: Figure S1. Chromatograms of the sequencing results. Chromatograms of the sequencing results of *ESCO2* gene showing both mutations: (A) The mutation (c.954_955 + 2delAAGT): a 4-bp deletion overlapping the end of exon 4 and the beginning of intron 4. (B) The mutation (c.1131 + 1G > A): a substitution of an Adenine for a Guanine base, a splice-site mutation in intron 6.
