# Supplementary figures and images for: 3D-FISH analysis reveals chromatid cohesion defect during interphase in Roberts syndrome
Source: Mol Cytogenet. 2014 Sep 30;7:59. doi: 10.1186/s13039-014-0059-6 (PMC4197286; doi:10.1186/s13039-014-0059-6)

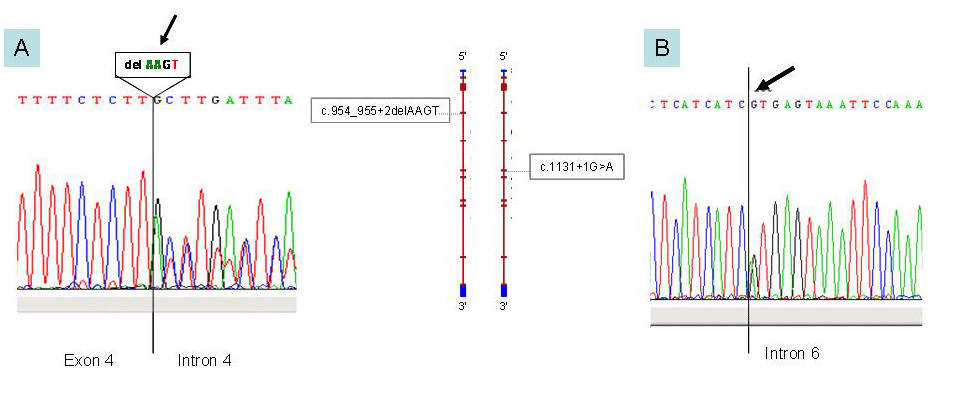

Supplement: Additional file 1: Figure S1. — Chromatograms of the sequencing results. Chromatograms of the sequencing results of ESCO2 gene showing both mutations: (A) The mutation (c.954_955 + 2delAAGT): a 4-bp deletion overlapping the end of exon 4 and the beginning of intron 4. (B) The mutation (c.1131 + 1G > A): a substitution of an Adenine for a Guanine base, a splice-site mutation in intron 6. [file 13039_2014_59_MOESM1_ESM.jpeg]
